# Isolated optic neuritis with a concurrent abnormal trigeminal nucleus on imaging: case report of a rare complication of herpes zoster ophthalmicus

**DOI:** 10.1186/s12883-018-1168-3

**Published:** 2018-10-04

**Authors:** Kavin Vanikieti, Anuchit Poonyathalang, Panitha Jindahra, Piyaphon Cheecharoen, Patchalin Patputtipong, Tanyatuth Padungkiatsagul

**Affiliations:** 1 Department of Ophthalmology, Faculty of Medicine Ramathibodi Hospital, Mahidol University, 270 Rama VI Road, Bangkok, 10400 Thailand; 2 Department of Medicine, Faculty of Medicine Ramathibodi Hospital, Mahidol University, 270 Rama VI Road, Bangkok, 10400 Thailand; 3 Department of Radiology, Faculty of Medicine Ramathibodi Hospital, Mahidol University, 270 Rama VI Road, Bangkok, 10400 Thailand

**Keywords:** Herpes zoster ophthalmicus, Optic neuritis, Magnetic resonance imaging, Diffusion weighted imaging, Trigeminal nucleus

## Abstract

**Background:**

Herpes zoster ophthalmicus (HZO) is an inflammation related to reactivation of the latent varicella zoster virus (VZV), involving the ophthalmic branch of the trigeminal nerve. Optic neuritis (ON), a rare ocular complication following HZO, has been reported in 1.9% of HZO-affected eyes. Most previous cases occurred simultaneously with other ocular complications, especially orbital apex syndrome. Moreover, detailed magnetic resonance imaging (MRI) with diffusion weighted imaging of the optic nerve and trigeminal nucleus in HZO-related ON has been rarely reported. We report a case of postherpetic isolated ON with a concurrent abnormal trigeminal nucleus on imaging.

**Case presentation:**

A healthy 58-year-old female presented with sudden painful visual loss in her right eye for 2 days. Four weeks before the presentation, her right eye was diagnosed with HZO, and she received intravenous acyclovir for 10 days. Ophthalmic examination revealed a visual acuity of light perception and 20/20 in the right and left eyes, respectively. A relative afferent pupillary defect was present in the right eye. Neurological examination was significant for hypoesthesia in the area of the HZO. A clinical diagnosis of HZO-related right retrobulbar ON was made, and other causes of atypical ON were excluded. MRI showed enhancement and restricted diffusion of the right-sided optic nerve with linear hyperintense T2 of the right-sided spinal trigeminal nucleus and tract (STNT) along the brainstem. She received 14 days of intravenous acyclovir and 5 days of methylprednisolone. Both were switched to an oral route for 2 months. After the completion of treatment, the visual acuity was counting fingers and 20/20 in the right eye and left eye, respectively. Stable brainstem STNT abnormalities and resolution of ON were found radiologically.

**Conclusions:**

Isolated ON is a rare ocular complication following HZO. An abnormal high signal of STNT on a T2 weighted image may be present, which may be a clue for VZV-associated complications, such as HZO-related ON, especially in cases lacking an obvious history of HZO or other concomitant ocular complications. Prompt treatment with both acyclovir and corticosteroids should be started. Restricted diffusion of the optic nerve may be a predictor for poor visual recovery.

## Background

Herpes zoster ophthalmicus (HZO) is an inflammation related to reactivation of the latent varicella zoster virus (VZV) involving the ophthalmic branch of the trigeminal nerve [1]. Ocular complications are found in 50% of HZO patients, following the onset of a rash in 1–4 weeks [2].

Any ocular structures can be affected, although anterior uveitis and keratitis are the most common ocular complications [3]. Optic neuritis (ON), a rare ocular complication following HZO, has been reported in 1.9% of HZO-affected eyes [3]. However, HZO-related ON in many previous reports was not isolated. Most of the previous cases occurred simultaneously with other ocular complications, especially orbital apex syndrome [4, 5]. Moreover, detailed magnetic resonance imaging (MRI) with diffusion weighted imaging (DWI) of the optic nerve and trigeminal nucleus in HZO-related ON has been rarely documented. Herein, we report a case of postherpetic isolated ON with a concurrent abnormal trigeminal nucleus on imaging.

## Case presentation

**Fig. 1:**
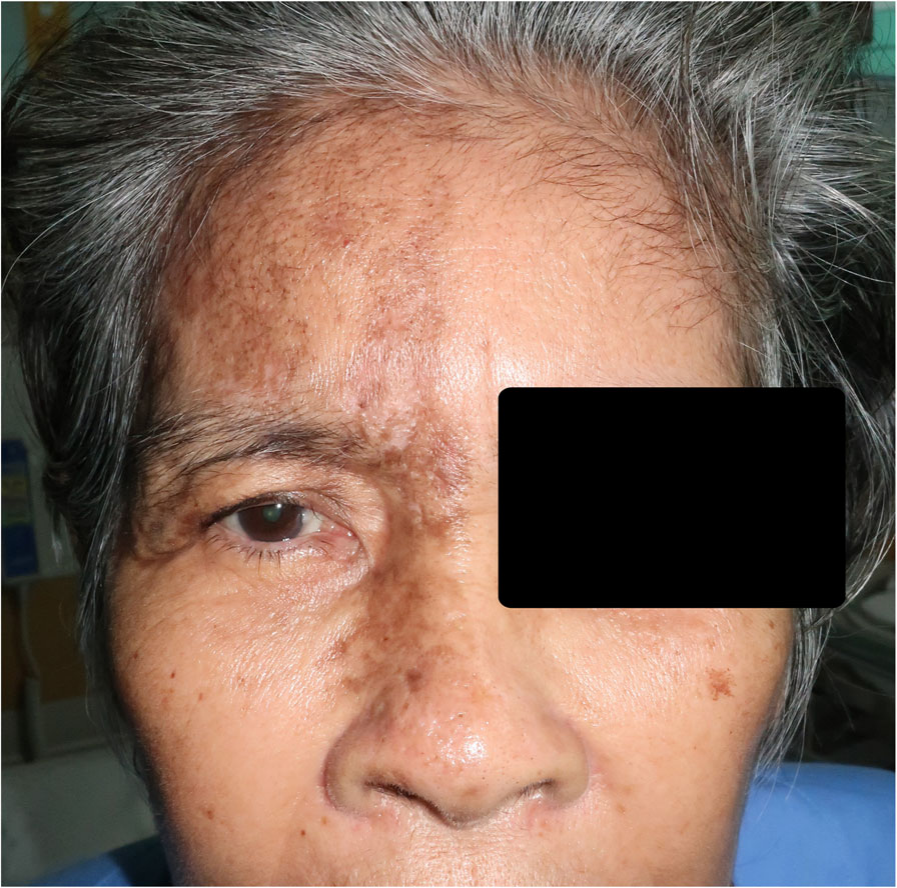
External appearance. Hyperpigmented macules and patches over the right ophthalmic branch of the trigeminal nerve, including the tip of the nose

A previously healthy 58-year-old female presented to our clinic with a sudden painful visual loss in her right eye for 2 days. Ocular movement significantly aggravated her pain. Four weeks before the presentation, she developed a group of vesicles on the erythematous base over the right ophthalmic branch of the trigeminal nerve including the tip of her nose, which was diagnosed as HZO. At that time, she was treated with intravenous acyclovir (30 mg/kg/day) for 10 days. The group of vesicles soon disappeared and turned to hyperpigmented macules and patches (Fig. 1).

**Fig. 2:**
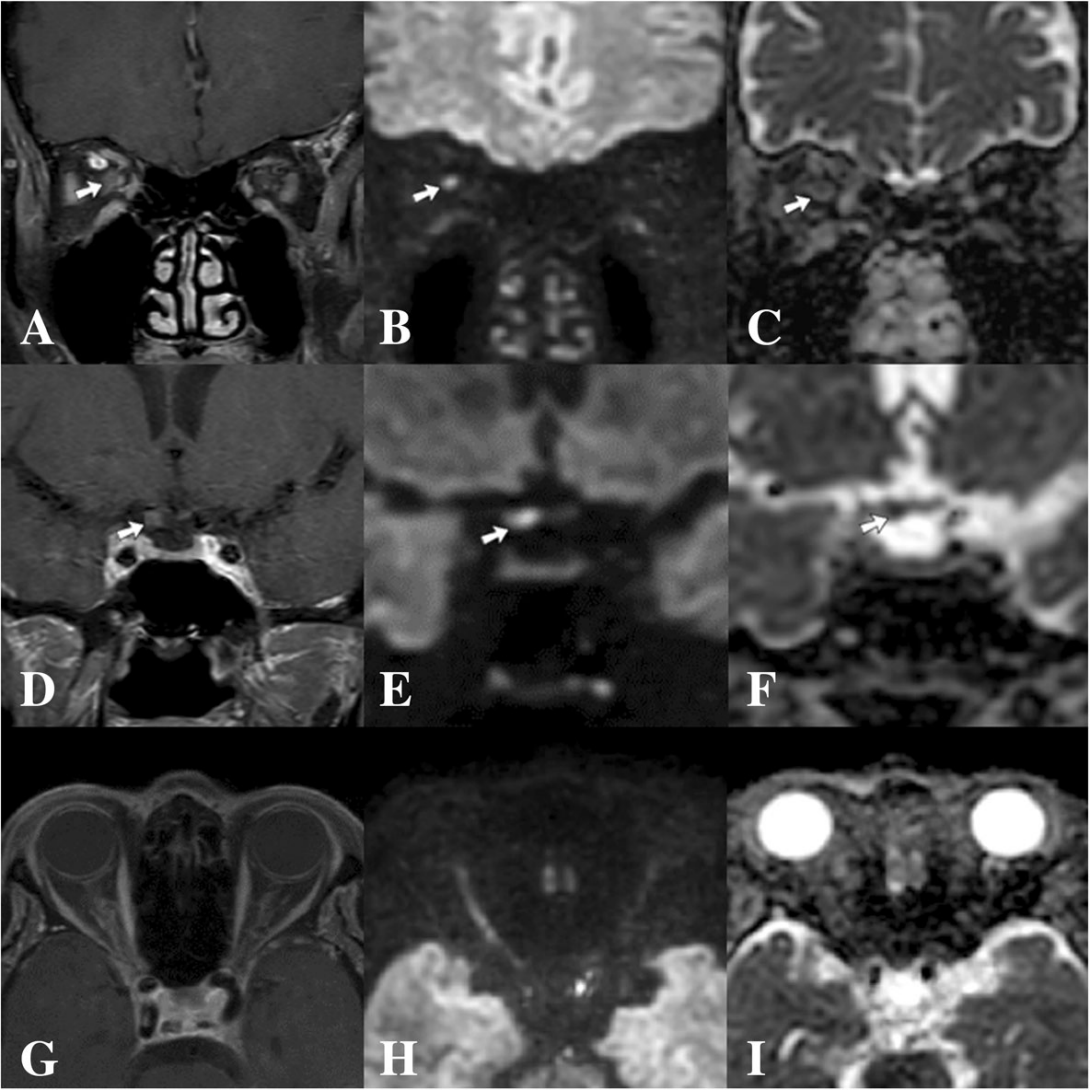
Magnetic resonance imaging of the orbit with diffusion weighted imaging (DWI) and an apparent diffusion coefficient (ADC). Coronal T1 weighted with a gadolinium injection (**a**) shows enhancement of the right intraorbital optic nerve (arrow) along with restricted diffusion (arrow) on DWI (**b**), which was confirmed by the low signal (arrow) on the ADC map (**c**). Coronal T1 weighted with a gadolinium injection (**d**) shows enhancement of the right prechiasmatic optic nerve (arrow) along with restricted diffusion (arrow) on DWI (**e**), which was confirmed by the low signal (arrow) on the ADC map (**f**). Axial T1 weighted with a gadolinium injection (**g**) shows enhancement of the right optic nerve along with restricted diffusion on DWI (**h**), which was confirmed by the low signal on the ADC map (**i**)

**Fig. 3:**
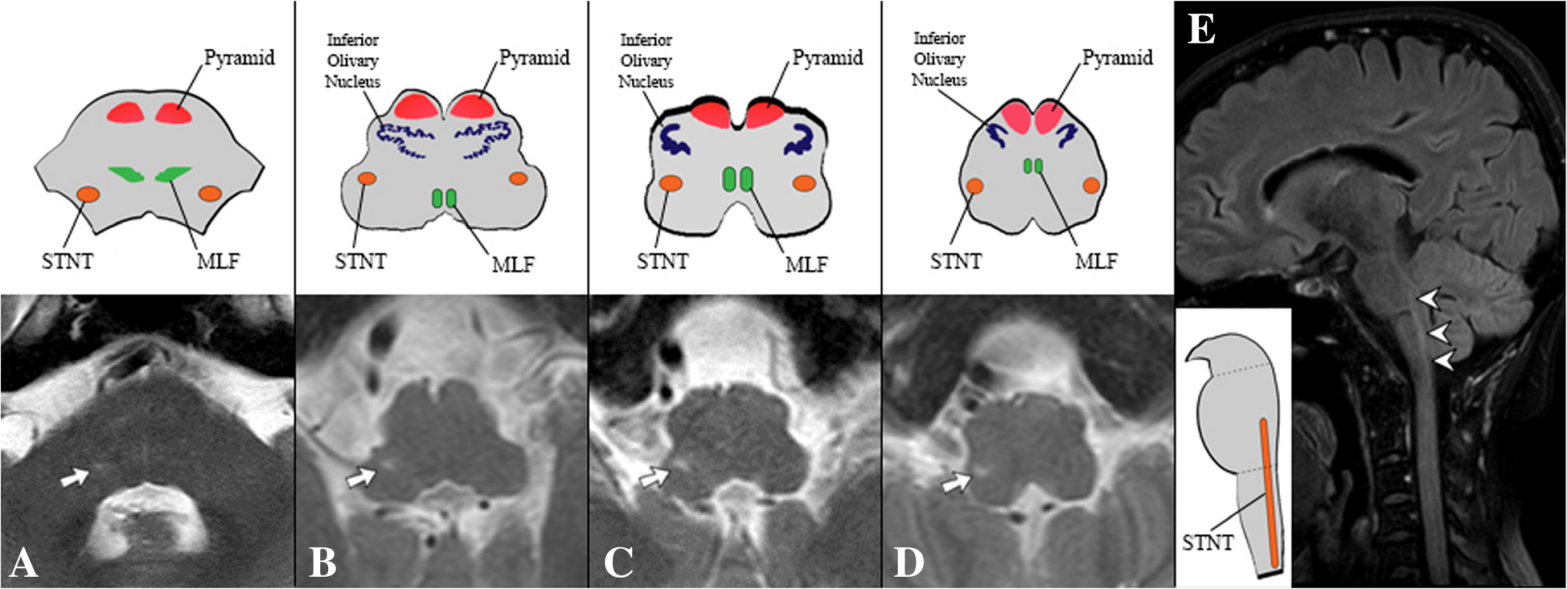
Magnetic resonance imaging of the brain*.* Axial T2 weighted shows hyperintense T2 lesions (arrows) extending from right dorsolateral pons to the medulla (**a**-**d**). Sagittal fluid attenuation inversion recovery (FLAIR) shows linear, vertical-oriented hyperintense lesions (arrowheads) (**e**). These lesions represent the anatomical location of the spinal trigeminal nucleus and tract (STNT) along the brainstem. MLF, medial longitudinal fasciculus

At our clinic, an ophthalmic examination revealed best-corrected visual acuity of light perception in the right eye, compared with 20/20 in the left eye. A relative afferent pupillary defect (RAPD) was present in the right eye. Intraocular pressures were 12 mmHg in both eyes. Ocular motility, anterior segment, and a fundus examination were unremarkable bilaterally. Neither proptosis nor ptosis was observed. The neurological examination was significant for hypoesthesia in the area supplied by the right ophthalmic branch of the trigeminal nerve. A clinical diagnosis of HZO-related right retrobulbar ON was made. To exclude other possible causes of atypical ON, a blood test including a complete blood count (CBC), erythrocyte sedimentation rate (ESR), c-reactive protein (CRP), Venereal Disease Research Laboratory (VDRL), *Treponema pallidum* hemagglutination (TPHA), antinuclear antibody (ANA), and aquaporin 4-antibody were performed, which all showed normal results. MRI of the brain and orbit showed enhancement and restricted diffusion of a right-sided intraorbital, intracanalicular, and prechiasmatic optic nerve (Fig. 2). Notably, linear hyperintense T2 lesions in vertical orientation extending from the right dorsolateral pons down to the medulla without any enhancement or restricted diffusion were also found (Fig. 3). These vertical lesions represented the anatomical location of the spinal trigeminal nucleus and tract (STNT) along the brainstem. Lumbar puncture showed mild lymphocytic pleocytosis (22 cells, 98% lymphocytes) with normal protein and a negative polymerase chain reaction (PCR) for VZV.

Treatment was started with intravenous acyclovir (30 mg/kg/day) along with 1 g/day of intravenous methylprednisolone. Intravenous acyclovir was continued for 14 days, then reduced to 800 mg oral acyclovir daily. Acyclovir was discontinued in the third month. Oral prednisolone (1 mg/kg/day) was started after 5 days of intravenous methylprednisolone, then gradually tapered and discontinued in the third month. After the completion of the 2 month treatment, the best-corrected visual acuity was counting fingers and 20/20 in the right and left eyes, respectively. An ophthalmic examination detected a right optic disc atrophy with normal physiological cupping. MRI of the brain and orbit showed stable brainstem STNT abnormalities and resolution of the ON.

## Discussion and conclusions

ON is an unusual ocular complication secondary to HZO that may present either in papillitis or retrobulbar form and occur weeks to months after the onset of the rash [6–12]. The degree of visual loss varies, ranging from mild to severe visual impairment [6–12]. Adults, especially the elderly, are more affected; however, HZO-related ON in children has also been reported [9, 13]. HZO-related ON in many previous reports was found simultaneously with other ocular complications, such as anterior uveitis, keratitis, and secondary glaucoma. Moreover, it may occur as part of the orbital apex syndrome [4, 5]. Our case uniquely demonstrated an isolated HZO-related ON without any concomitant ocular complications, which has been rarely reported.

Despite the absence of VZV in the cerebrospinal fluid (CSF) using the PCR, a diagnosis of HZO-related ON was made based on the following:

1. The concordance in laterality between the HZO and ON.
2. Nasociliary nerve involvement of HZO based on the presence of hyperpigmented macules and patches over the tip of the nose, which was highly associated with the development of ocular complications [2].
3. A temporal relationship between the HZO and ON.
4. A high signal of STNT on the T2 weighted MRI.
5. The exclusion of other possible causes of atypical ON.

The precise mechanism of ON following HZO is controversial. Viral replication in the ophthalmic branch of the trigeminal nerve, located in the cavernous sinus, spreads through the superior orbital fissure to the orbit, where it may cause direct injury to the optic nerve [14]. Extensive inflammation involving posterior ciliary arteries and nerves based on histopathology of HZO-affected eyes, which may lead to ocular ischemia and subsequent optic nerve damage, is another possible mechanism [15]. Parainfectious ON, as a consequence of a self-immune response triggered by the VZV antigen, has also been postulated [16]. These proposed mechanisms are usually difficult to differentiate from each other.

MRI findings of the optic nerve in HZO-related ON have been rarely described. Peripheral enhancement of the intraorbital optic nerve sheath has been commonly reported [4, 5, 12]. In our case, not only the optic nerve sheath, but also the axial portion of the optic nerve itself was affected. Moreover, the enhancement extended to the intracanalicular and prechiasmatic optic nerve. These results are consistent with the results of Wang et al., suggesting that these findings explained the severe visual impairment of ON and poor visual outcome in our case [12]. To our knowledge, DWI findings of the optic nerve have never been reported in HZO-related ON. However, restriction of the optic nerve on DWI, which is consistent with infarction attributed to compression or inflammation of the vessels serving the optic nerve, are rarely present in ON [17, 18]. Our case demonstrated a very long restricted diffusion of the optic nerve, including the entire intraorbital segment extending to the prechiasmatic segment. This may be a predictor for poor visual recovery in our case.

We found an unusually high signal of STNT on the T2 weighted MRI. This is thought be a result of VZV migration from the gasserian ganglion to the STNT along the brainstem [5, 19–21]. Douglas et al. reported contiguous hyperintense T2 lesions with restricted diffusion on DWI along the brainstem, which corresponded to the anatomical location of STNT in a VZV encephalitis patient [21]. Two months following complete anti-viral treatment, these lesions improved [21]. In our case, STNT failed to show any restriction on DWI. However, the restriction of the right-sided optic nerve observed in our case might be compatible with the restricted diffusion of the STNT reported by Douglas et al. [21].

Because the proposed mechanism of HZO-related ON is poorly understood and more than one mechanism can occur, it is reasonable to start the treatment with a combination of acyclovir and corticosteroids. In many previous reports, the duration of treatment varied, ranging from 10 days to 2 months [6–12]. We decided to continue the treatment up to 2 months because of the severe visual impairment of ON.

Visual recovery in ON following HZO is typically excellent [6–12]. Nevertheless, severe visual outcomes have also been reported [3]. Concomitant ocular complications, such as retinitis and secondary glaucoma may contribute to a poor visual prognosis. Based on the MRI with DWI of the optic nerve, we hypothesized that poor visual recovery in our case was due to the following:

1. Both axial and optic nerve sheath portions were affected.
2. A very long enhancement with restricted diffusion of the optic nerve occurred.

In summary, we report a case of isolated ON following HZO along with restricted diffusion of the optic nerve on imaging. In addition, an abnormal high signal of STNT on the T2 weighted image was found. This may be a clue of VZV-associated complications, such as HZO-related ON, especially in cases lacking an obvious history of HZO or other concomitant ocular complications. Prompt treatment with both acyclovir and corticosteroids should be started. Restricted diffusion of the optic nerve may therefore be a predictor for poor visual recovery.

## Abbreviations

ANA: Antinuclear antibody
CBC: Complete blood count
CRP: C-reactive protein
CSF: Cerebrospinal fluid
DWI: Diffusion weighted imaging
ESR: Erythrocyte sedimentation rate
HZO: Herpes zoster ophthalmicus
MLF: Medial longitudinal fasciculus
MRI: Magnetic resonance imaging
ON: Optic neuritis
PCR: Polymerase chain reaction
RAPD: Relative afferent pupillary defect
STNT: Spinal trigeminal nucleus and tract
TPHA: *Treponema pallidum* hemagglutination
VDRL: Venereal Disease Research Laboratory
VZV: Varicella zoster virus
